# 4091 Influence of Vision and Proprioception on Motor Control in ASD

**DOI:** 10.1017/cts.2020.303

**Published:** 2020-07-29

**Authors:** Robin L Shafer, Zheng Wang, Matthew W. Mosconi

**Affiliations:** 1 University of Kansas; 2 University of Florida

## Abstract

OBJECTIVES/GOALS: Sensorimotor integration deficits are common in Autism Spectrum Disorders (ASD). There is evidence for both an over-reliance on visual and proprioceptive feedback during motor control in ASD, suggesting deficits in the ability to modulate sensory feedback processing in order to use the most reliable input. This study aims to test this hypothesis. METHODS/STUDY POPULATION: 40 persons with ASD (ages 10-33 yrs) and 25 age-, sex- and nonverbal IQ-matched controls completed precision gripping tasks under multiple proprioceptive and visual feedback conditions. Participants squeezed a force sensor with their index finger and thumb and tried to match their force output to a target force. Visual feedback of the target force (stationary bar) and their force output (bar that moved up/down with increased/decreased force) were displayed on a computer screen. Visual feedback was presented across low, medium, and high gain levels; the force bar moved a greater distance per change in force at higher gains. Proprioceptive feedback was manipulated using 80Hz tendon vibration at the wrist to create an illusion that the muscle is contracted. Force regularity (approximate entropy; ApEn) was examined. RESULTS/ANTICIPATED RESULTS: We have scored data from 18 participants with ASD and 13 control participants to date. Preliminary results from these participants indicate a Group x Tendon Vibration x Visual Gain interaction for ApEn (F = 1.559, p = 0.023). Individuals with ASD show slight increases in ApEn with 80Hz tendon vibration relative to no tendon vibration in all visual conditions. Controls showed increased ApEn during 80Hz compared to no tendon vibration at low visual gain but decreased ApEn with tendon vibration at high visual gain. These preliminary results indicate that controls shift to using a secondary source of sensory feedback (e.g., proprioception) when the primary source (e.g., vision) is degraded. However, persons with ASD do not reweight different sensory feedback processes as feedback inputs are degraded or magnified. DISCUSSION/SIGNIFICANCE OF IMPACT: Our preliminary results reveal that sensorimotor issues in ASD result from deficits in the reweighting of sensory feedback. Namely, persons with ASD fail to dynamically recalibrate feedback processes across visual and proprioceptive systems when feedback conditions change. Our results may aid treatment development for sensorimotor issues in ASD.

